# Identification of *Hafnia alvei* by MALDI-TOF MS and Their Antimicrobial Resistance Profiles from Milk of Dairy Cows with Subclinical Mastitis

**DOI:** 10.3390/microorganisms14040741

**Published:** 2026-03-26

**Authors:** Khasapane George Ntelekwane

**Affiliations:** Department of Life Science, Central University of Technology, Bloemfontein 9300, Free State, South Africa; nkhasapane@cut.ac.za

**Keywords:** intramammary infection, *Enterobacteriaceae*, MALDI-TOF MS, dairy cattle, raw milk

## Abstract

*Hafnia alvei*, which belongs to the *Enterobacteriaceae* family, has been occasionally documented in animal infections but is still not well characterized in the context of bovine mastitis. This research examined the prevalence and antimicrobial resistance characteristics of *H. alvei* in dairy cows suffering from subclinical mastitis in South Africa’s Free State Province. In the Thabo Mofutsanyana District, a total of 174 milk samples were obtained from cows on six different dairy farms. The California Mastitis Test (CMT) was used to screen for subclinical mastitis, and somatic cell count was used to confirm it. Standard culture methods were used for bacterial isolation, and presumptive *Enterobacteriaceae* isolates were identified through matrix-assisted laser desorption/ionization time-of-flight mass spectrometry (MALDI-TOF MS). Out of the 174 samples, 84 (48.2%) tested positive for CMT, and 68 (39.1%) met the SCC criteria for subclinical mastitis at a cow level, while 96/336 (28.5%) were infected at a quarter level. Of the 100 presumptive *Enterobacteriaceae* isolates, 33 (33.0%) were identified as *H. alvei* (*p* = 0.0034). Antimicrobial susceptibility testing showed that 50% of the isolates were resistant to penicillin, followed by tetracycline and erythromycin with 25% and 10%, respectively. Furthermore, the results showed that 17 (51.5%) isolates exhibited multidrug-resistant profiles. The results suggest that *H. alvei* could be a contaminant in raw milk associated with bovine subclinical mastitis in this area, necessitating additional epidemiological research that includes healthy matched controls.

## 1. Introduction

*Hafnia alvei* Møller, a psychotropic member of the *Enterobacteriaceae* family, is often found in raw milk, seafood, and chilled meats, serving primarily as a spoilage organism [1]. *Hafnia alvei* has been found in some instances of bovine mastitis and is considered an environmental opportunistic intramammary pathogen [2]. Although it has the potential to cause disease due to biofilm formation and inflammation caused by endotoxins, its significance in epidemiology is much less than that of major mastitis pathogens. According to recent studies, its spoilage activity is governed by a LuxI/LuxR-type quorum-sensing (QS) system that regulates biofilm formation, motility, and enzyme production [3,4]. Biofilm development and the formation of spoilage metabolites are greatly diminished when QS is disrupted, and this effect can be lessened by natural quorum-quenching substances like theaflavins [5]. Human infections are uncommon, with isolated cases such as granulomatous mastitis [6], reinforcing its categorization as a rare opportunistic pathogen [5,6]. In dairy environments, *H. alvei* often coexist with foodborne pathogens like *Escherichia coli*, *Salmonella enterica*, *Listeria monocytogenes*, and *Staphylococcus aureus* [7]. According to Irlinger et al. [7], interactions between *H. alvei* and these pathogens are frequently facilitated by competitive exclusion mechanisms, which involve competition for nutrients, iron, and ecological niches on milking equipment and food-contact surfaces. Due to its psychrotolerant characteristics, *H. alvei* can grow at refrigeration temperatures. This allows it to survive and even become a dominant organism in the microbial populations of raw milk during cold storage, possibly restricting the growth of more temperature-sensitive pathogens [6].

Aside from competition, various studies have shown that *H. alvei* strains can generate inhibitory metabolites or substances resembling bacteriocins that inhibit pathogenic bacteria, especially *Listeria monocytogenes* and other Enterobacterales [5]. Moreover, *H. alvei* have quorum-sensing systems, such as AI-2-mediated signaling, that could disrupt interspecies communication and influence virulence gene expression and biofilm formation in co-existing pathogens [8].

A group of goats with different severity levels of pneumonia was examined by Khan et al. [9], who noted that 9.83% of the samples tested positive for this bacterium. Mastitis in cattle is a prevalent cause of significant economic losses in Turkish dairies [10], with global estimates exceeding 28 million USD [11]. It has been shown that various bacteria are the primary cause of mastitis. Their epidemiological association further classified bacterial agents into environmental and contagious pathogens [1,12]. Foodborne diseases play a crucial role in the One Health framework promoted by the World Health Organization, stemming from the interconnections among human, animal, and environmental health [13]. Managing this necessitates integrated and multisectoral approaches to lower pathogen transmission, address antimicrobial resistance, guarantee food safety, and bolster global health security [14].

Consequently, quick identification of pathogens is crucial to diminishing the risk of their dissemination before the outbreak of an epidemic. Various techniques have been devised to enhance the methods used for its detection. Microbiology can be investigated using traditional and chemical approaches or through molecular biology techniques such as polymerase chain reaction technology [15,16]. Furthermore, the phenotypic characterization of *H. alvei* strains obtained from raw milk shows that they possess multidrug resistance traits. According to research conducted by Yi et al. [17], isolates from raw milk demonstrated resistance to a wide variety of antibiotics, such as macrolides (e.g., midecamycin and clarithromycin), clindamycin, oxacillin, cephalothin, and cefazolin. However, they showed moderate susceptibility to tetracycline, erythromycin, and certain cephalosporins.

It is for these reasons that the current study aimed to investigate the prevalence of *H. alvei* isolates from milk of subclinical mastitis dairy cows in the Free State Province, South Africa, using MALDI-TOF MS, and further determine their antimicrobial resistance statuses using the disk diffusion method. In addition, the hypothesis was put forward that MALDI-TOF MS is a reliable method for identifying *H. alvei* isolates in milk samples. It was further hypothesized that the *H. alvei* isolates identified exhibit different antimicrobial resistance patterns when evaluated with the disk diffusion method.

## 2. Materials and Methods

### 2.1. Sampling and Mastitis Screening

**Figure 1:**
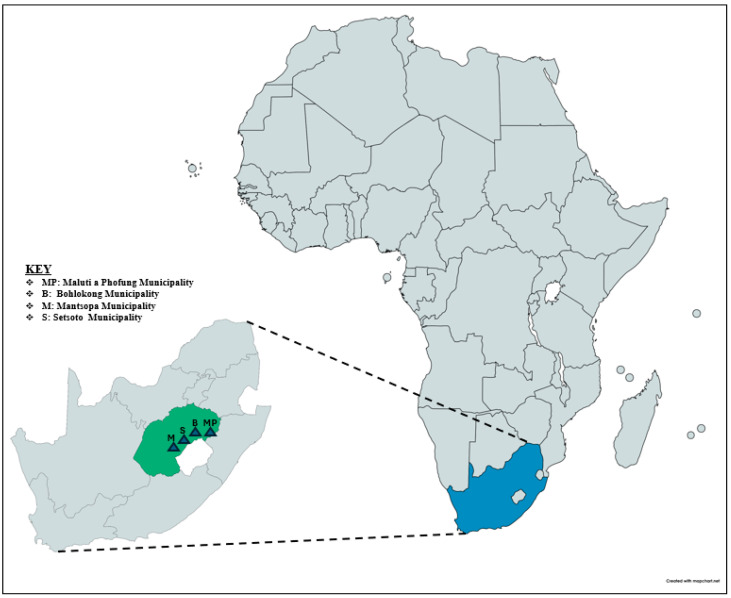
Geographical locations of sample collection sites within the Free State Province, South Africa.

The current cross-sectional study was conducted in March–June 2024 in the Thabo Mofutsanyana District Municipality of Free State Province (Figure 1) across six semi-intensive farms. From four local municipalities—Mantsopa (40), Bohlokong (40), Maluti-a-Phofung (40), and Setsoto (54)—milk samples were collected using 50 mL sterile containers. Aseptically collected milk samples were taken from each (4) quarter of cows that looked healthy, in accordance with the National Mastitis Council (NMC) guidelines. Cow level information—Age (2 categories: ≤4 y versus >4 y), parity (primiparous versus multiparous), breed, and lactation stage [3 categories: 180 DIM] was recorded for every cow. Quarter-level information—For each quarter, position (4 categories: left front, left hind, right hind, right front) and presence of teat lesions (2 categories: yes, versus no) were recorded

The California Mastitis Test (CMT) was used to screen a cow for subclinical mastitis on the farm. To prevent cross-contamination, each cow’s udder was cleaned with distilled water and dried with a disposable paper towel before sampling. A total of 174 composite milk samples from individual cows were randomly screened for intramammary infection by means of somatic cell count (SCC) assay using flow cytometry (Mérieux NutriSciences, Cape Town, South Africa). Thereafter, based on the SCC results, only 696 individual quarters from 84 cows with IMI based on SCC to the California mastitis test (CMT) according to manufacturer’s instructions (DeLaval, Bloemfontein, South Africa) on farm, briefly, 10 mL of milk was collected from each lactating quarter free of clinical mastitis and placed into separate cups on the CMT paddle. The milk samples were equalized by tilting the CMT paddle and decanting the extra milk. An equal quantity of CMT reagent was added to each cup of the CMT paddle that held the milk, and this mixture was thoroughly combined for 15 s, and subsequently, only 58 quarter milk samples were collected for another round of SCC and microbiological analysis on the same day.

Thereafter, positive cow samples were then checked for SCM using the somatic cell count (SCC) assay (Mérieux NutriSciences, South Africa), when a cow was classified as having subclinical mastitis. The SCC results were evaluated and categorized as a healthy quarter if the SCC was ≤100,000 cells/mL milk, a weak positive quarter if it fell between >100,000 and <500,000 cells/mL milk, a distinct positive if it was >500,000 and <1,000,000 cells/mL milk, and a strong positive if SCC was ≥1,000,000 cells/mL milk, following the recommendations of Karzis et al. [18] and Khasapane et al. [19].

### 2.2. Bacterial Isolation

Milk samples (10 μL) were streaked onto Harlequin^®^ *E. coli*/Coliform Agar (Neogen, Lansing, MI, USA) and incubated for 24 h at 37 °C. On the other hand, samples that isolated more than two bacterial species were deemed tainted. Colonies with the same chromogenic characteristics (pinkish colonies) were reisolated on nutrient agar (Neogen, Lansing, MI, USA), and single colonies were sequentially transferred to create a pure culture [20].

### 2.3. Bacterial Identification by MALDI-TOF MS

The identification of isolates was performed with the Biotyper 3.1 software (Bruker, Johannesburg 2191, South Africa). To calibrate the Autoflex Speed equipment (Bruker Daltonics, Billerica, MA, USA), the *Escherichia coli* DH5α Bacterial Test Standard (BTS) was utilized. Moreover, all bacterial isolates were identified following the method of Cameron et al. [21,22]. Using sterile disposable plastic inoculating loops of 1.0 μL, the pure colony was briefly transferred to separate Safe-Lock Tubes (Sigma-Aldrich, Brondbyvester, Denmark) of 1.5 mL each that contained 300 μL of ultra-HPLC grade water (Merck, Hellerup, Denmark). After Safe-Lock Tubes were vortexed briefly to achieve a homogenous suspension, 900 μL of 100% ethanol (Merck) was added and vortexed for another 15 s. Safe-Lock Tubes were centrifuged for 3 min at 12,200× *g* at room temperature (RT). The supernatants were poured out and discarded, and the Safe-Lock Tubes underwent a second centrifugation for 3 min under the same conditions. The remaining ethanol/water was carefully aspirated using a micropipette. Before adding 70% formic acid in an appropriate volume (15–50 μL), bacterial cell pellets were air-dried for 3 min. The ideal amount of formic acid was found by visually assessing the size of the pellet. After a maximum of 3 min, each sample received an equal volume of 100% acetonitrile, which was then mixed with care. At room temperature, samples were spun in a centrifuge for 3 min at 12,200× *g*. Eight 1-μL aliquots of supernatant were meticulously positioned on a ground steel target plate and permitted to air dry. After drying, each spot was covered with 1.0 μL of a solution containing cyano-4-hydroxycinnamic acid (HCCA) matrix (Bruker Daltonics) in 50% acetonitrile and 2.5% trifluoroacetic acid (Sigma-Aldrich). Each isolate was analyzed in duplicate. If isolates were not resolved after two rounds of MALDI-TOF MS analysis, they were declared unidentified. To ensure the reliability of our analysis, we established a cut-off score of ≥2.0 as the threshold for identifying bacteria.

### 2.4. Phenotypic Antimicrobial Resistance

Antimicrobial susceptibility was assessed using the disc diffusion method on Mueller–Hinton agar (Merck, Darmstadt, Germany). The discs were put on a plate and incubated aerobically for 18 to 24 h at 37 °C following spreading the overnight inoculum containing the isolates. The results were interpreted according to the VET01-S: Performance Standards for Antimicrobial Susceptibility Testing by the Clinical and Laboratory Standards Institute [23]. Six antibiotics were used in this study, including ampicillin (10 μg), ciprofloxacin (5 μg), erythromycin (15 μg), gentamicin (10 μg), penicillin (10 μg) and tetracycline (30 μg) purchased from Thermo Fischer Scientific™ (Thermo Fisher Scientific, Waltham, MA, USA) (Table 1). Moreover, *H. alvei* ATCC 51815 (Thermo Fisher Scientific, Waltham, MA, USA) was used as a positive control in this study.

### 2.5. Statistical Analysis

Data were input into Microsoft Excel and then analysed with IBM SPSS Statistics for Windows, version 26.0 (IBM Corp., Armonk, NY, USA). The data were summarized using descriptive statistics. To check whether the species are normally distributed across the municipalities, a Chi-Square Goodness-of-Fit Test was conducted. Results were deemed statistically significant at a *p*-value of less than 0.05, and all statistical tests were conducted as two-tailed.

## 3. Results

### 3.1. California Mastitis Test (CMT) and Somatic Cell Count (SCC)

Of the 174 sampled cows, CMT results indicated that 84 (48.2%) had signs of intramammary infection. Moreover, at the cow level based on SCC, only 68 (39.1%) of these cows tested positive for subclinical mastitis. Thereafter, the quarter sample results showed that 96/336 (28.5%) of the quarters were subclinically infected by mastitis.

### 3.2. Identification of H. alvei

A total of 171 Enterobacteriaceae strains were isolated from 174 milk samples collected from dairy cows with subclinical mastitis on six farms. From these, 71 have already been published from our previous study [21]. Furthermore, for the current study, MALDI-TOF-MS analysis confirmed the identification of all 33/100 (33%) were *Hafnia alvei* strains (Supplementary File S1). The *p*-value (0.0034) being below 0.05 indicates a significant difference between the observations’ distribution and an even distribution of isolates across the four municipalities. The highest percentage of species was isolated from Maluti-a-Phofung (16%), followed by Bohlokong (10%), Setsoto (5%), and Mantsopa (2%) local municipality.

### 3.3. Antimicrobial Resistance

**Figure 2:**
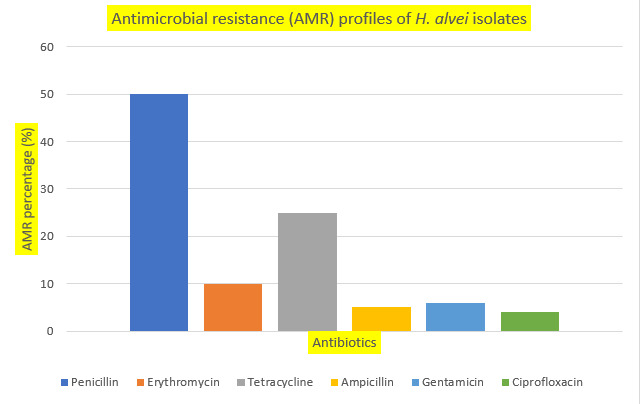
Phenotypic antimicrobial resistance profiles of *H. alvei* strains, where AMR stands for antimicrobial resistance.

According to the results of the disc diffusion test, the majority of the *H. alvei* isolates were resistant to penicillin (50%), followed by tetracycline and erythromycin at 25% and 10%, respectively (Figure 2). The isolates were least resistant to gentamicin, ampicillin, and ciprofloxacin, respectively. Furthermore, the results of a chi-square test indicated a significant association between the type of antibiotic and resistance status (χ^2^ = 114.96, df = 5, *p* < 0.0001), suggesting that resistance patterns varied among the antibiotics examined.

### 3.4. Multi-Drug Resistance

Furthermore, data showed that 17 (51.5%) isolates exhibited multidrug-resistant profiles. The majority of them, 6 (35.2%), were resistant to both Penicillin and Ciprofloxacin, while 2 (11.7%) of the isolates were resistant to Penicillin, Ciprofloxacin, Gentamycin, and Penicillin, Ciprofloxacin, and Tetracycline, respectively (Table 2).

## 4. Discussion

It is remarkable that *H. alvei* was detected in one third of *Enterobacteriaceae* isolates, considering that most bovine mastitis studies primarily concentrate on established pathogens like *Staphylococcus aureus Rosenbach*, coagulase-negative staphylococci, streptococci (e.g., *Streptococcus uberis Diernhofer*, *Streptococcus agalactiae Lehmann and Neumann*), and *Escherichia coli Miggula*, with little attention given to *Hafnia* species [24,25]. For instance, a meta-review on bovine mastitis in sub-Saharan Africa indicates that the average prevalence of subclinical mastitis is 15–50% across various studies (with clinical cases being less frequent) and enumerates the usual significant pathogens (such as *S. aureus* and *E. coli*), yet does not highlight *H. alvei* [26].

Another study of the milk microbiota from healthy and mastitic Sahiwal cattle (Pakistan) showed that Proteobacteria were predominant in the healthy and subclinical groups (56.48% and 48.77%, respectively), while their representation decreased in cases of clinical mastitis (2.68%) [27]. Even though that paper did not identify Hafnia at the species level, the prominence of Proteobacteria indicates that Gram-negative bacilli (including Hafnia) may be under-recognized in SCM contexts. A study conducted in Iraq in 2023 found that *H. alvei* was isolated from 20% of raw cow milk samples and 13.33% of raw buffalo milk samples [28]. The work titled “Screening commercial teat disinfectants against bacteria isolated from bovine milk” identified *H. alvei* as an environmental mastitis-associated isolate (one sample) found in bulk tank milk (BTM) and categorized among “environmental bacteria” [29].

Moreover, a study conducted in China in 2023 outlined the biological traits of *H. alvei* found in raw milk (resistance traits, motility, and biofilm), but this was also within the context of dairy milk and not specifically related to mastitis cases [30]. In summary, despite the isolation of *H. alvei* from dairy milk contexts, there is limited documentation of its direct link to bovine intramammary infection. Consequently, the discovery that 33% of *Enterobacteriaceae* isolates are *H. alvei* in SCM cases offers valuable new evidence backing the theory that this bacterium could be a potential mastitis pathogen—or at the very least, part of an udder-microbiome dysbiosis.

A strain of *H. alvei* (HY-1) isolated from raw milk was found to demonstrate swimming motility, biofilm formation, and tolerance of different levels of salt/acid/alkali in a Chinese study conducted in 2023 [29]. The formation of biofilm is an acknowledged characteristic that can aid in the persistence within lactating udder tissues or milking apparatus, enabling colonization and chronic low-grade infection [31]. Meanwhile, studies of raw milk from Iraq indicate that *H. alvei* may be present in the milk microbiota of apparently healthy animals, supporting the idea of it being an opportunist or latent colonizer rather than a primary pathogen [2]. Moreover, the identification of *H. alvei* in the teat-disinfectant screening study (within the environmental bacterial group) reinforces the idea that environmental exposure (from milking equipment and teat surfaces) may play a role in the introduction of *H. alvei* into the udder [27]. In light of our finding that *H. alvei* was present in 33% of *Enterobacteriaceae* isolates from SCM cows, it is possible that *H. alvei* serves as an emerging opportunistic intramammary pathogen in this context or as part of Gordian microbial communities that contribute to subclinical infection [1]. It is crucial to note that, by definition, subclinical mastitis is low-grade and characterized by an elevated SCC without any obvious clinical signs. This increases the likelihood of detecting non-traditional agents.

The antimicrobial susceptibility profile of the *Hafnia alvei* isolates showed significant variability among the tested antibiotics, indicating different resistance mechanisms and levels of antimicrobial exposure. The disc diffusion test revealed that penicillin exhibited the greatest resistance (50%), followed by tetracycline (25%) and erythromycin (10%). In contrast, most isolates were still susceptible to ampicillin, gentamicin, and ciprofloxacin. The results underscore the presence of both intrinsic and acquired resistance characteristics in *H. alvei*, calling into question the effectiveness of widely used antibiotics.

The significant resistance to penicillin aligns with earlier findings that characterize *H. alvei* as an Enterobacterales member with innate resistance to multiple β-lactam antibiotics [16]. According to Hardefeldt et al. [31], the common use of β-lactams in veterinary medicine, especially for mastitis treatment, could add to selective pressure and the persistence of resistant strains.

The moderate tetracycline resistance (25%) noted in this research corresponds with findings of tetracycline resistance in Gram-negative bacteria obtained from food-producing animals. Tetracycline resistance is often linked to efflux pumps and ribosomal protection proteins encoded by tet genes found on mobile genetic elements, which promote horizontal gene transfer [32]. It is especially worrying that *H. alvei* can contain such resistance determinants and can survive in a variety of environments, one of which is the dairy production chain.

Ten percent of the isolates showed resistance to erythromycin. Despite macrolides typically being less effective against Enterobacterales due to limited outer membrane permeability, there have been reports of acquired resistance mediated by *erm* genes in Hafnia species and other enteric bacteria [33]. Even low-level detection of erythromycin resistance indicates continuous antimicrobial selection and the possibility of co-resistance with other drug classes.

Conversely, the susceptibility rates for ampicillin, gentamicin, and ciprofloxacin were high, suggesting that these antibiotics are still largely effective against the isolates examined. According to Hardefeldt et al. [31], gentamicin’s low resistance can be linked to its limited use and parenteral administration, which minimizes indiscriminate exposure and decreases selective pressure. Ciprofloxacin showed the greatest susceptibility, aligning with earlier research that noted a relatively low resistance to fluoroquinolones among *H. alvei* isolates from animal sources, potentially linked to regulatory limitations on fluoroquinolone use in food-producing animals.

The differences in resistance patterns among the antibiotics that were statistically significant highlight the necessity of routine antimicrobial susceptibility testing before treatment. The presence of resistant *H. alvei* isolates in subclinical mastitis raises concerns from veterinary and public health perspectives, as this organism is increasingly recognized as an opportunistic pathogen that can cause infections in both animals and humans [32]. Additionally, bacteria resistant to antimicrobials that come from dairy herds can infiltrate the food chain, presenting a possible threat to consumers and adding to the worldwide issue of antimicrobial resistance [33].

From a diagnostic standpoint, the application of MALDI-TOF MS in our research is praiseworthy, as this method likely enhances species-level identification beyond what routine culture/biochemistry can provide [34,35]. This could be a partial reason for the fact that our detection rate of *H. alvei* is relatively high in comparison to older studies that may not have identified Hafnia at the species level. It highlights that the use of contemporary diagnostic tools can uncover organisms in mastitis that were not acknowledged before [36]. The AMR findings underscore the necessity for careful use of antimicrobials, ongoing monitoring of resistance trends, and the establishment of antimicrobial stewardship programs in dairy production systems. It is crucial to take such measures in order to maintain the effectiveness of current antibiotics and reduce the transmission of resistant *H. alvei* strains where animals and humans interact [37,38]. From a management standpoint, the potential involvement of *H. alvei* brings up inquiries regarding the choice of antimicrobial, the persistence of infection, and the possible implications for milk quality, integrity of udder tissue, and transmission risk through equipment or bulk tank. Thus, under the WHO’s ONE HEALTH framework, this *H. alvei* is a significant zoonotic pathogen for both humans and animals, despite its rarity. As a limitation of the study, due to the cross-sectional design of the study, it was not possible to conduct repeatability testing through repeated sampling. The data thus constitute a snapshot of the study population at a specific time, which restricts the evaluation of temporal variability. Additional epidemiological research that includes healthy control groups and molecular analysis of resistance mechanisms is needed. Furthermore, the results of risk factors such as lactation stage, age, parity and quarter-level information were not recorded for the current study.

## 5. Conclusions

The evidence presented in this study suggests that *Hafnia alvei* may be one of the pathogens linked to subclinical mastitis in dairy cows from South Africa’s Free State Province and shows significant resistance to widely used antimicrobial drugs. The results highlight the need for enhanced antimicrobial stewardship in dairy herds, which encompasses judicious use of antimicrobials, regular susceptibility testing, and ongoing surveillance of opportunistic pathogens.

## Figures and Tables

**Table 1:** Zone diameter breakpoints for *H*. alvei isolates.

Antibiotic Disks	Concentration (µg)	Abbreviations	Resistant (mm)	Intermediate (mm)	Susceptibility (mm)
Ampicillin	10	AMP	≤13	14–16	≥17
Ciprofloxacin	5	CIP	≤15	16–20	≥21
Erythromycin	15	E	≤13	14–22	≥23
Gentamicin	10	CN	≤12	13–15	≥16
Penicillin	10	P	≤28	……	≥29
Tetracycline	30	TET	≤11	12–14	≥16

**Table 2:** Multi-drug resistance amongst *H. alvei* isolates.

AMR Phenotypes	Number of Isolates (%)
P, CIP	6
P, CN	1
P, CIP, CN	2
P, CIP, AMP	1
P, CIP, TET	2
CN, CIP, E	1
P, CIP, CN, AMP	1
P, CIP, AMP, TET	1
P, CIP, AMP, E	1
P, CIP, CN, TET	1

P: Penicillin, CIP: Ciprofloxacin, CN: Gentamycin, AMP: Ampicillin, TET: Tetracycline, E: Erythromycin.

## Data Availability

The original contributions presented in this study are included in the article/Supplementary Materials. Further inquiries can be directed to the corresponding author.

## Supplementary Materials

The following supporting information can be downloaded at: https://www.mdpi.com/article/10.3390/microorganisms14040741/s1. File S1: The results from MALDI-TOF MS.
