# Supplementary figures and images for: Identification of Hafnia alvei by MALDI-TOF MS and Their Antimicrobial Resistance Profiles from Milk of Dairy Cows with Subclinical Mastitis
Source: Microorganisms. 2026 Mar 26;14(4):741. doi: 10.3390/microorganisms14040741 (PMC13119061; doi:10.3390/microorganisms14040741)

## Slide 1
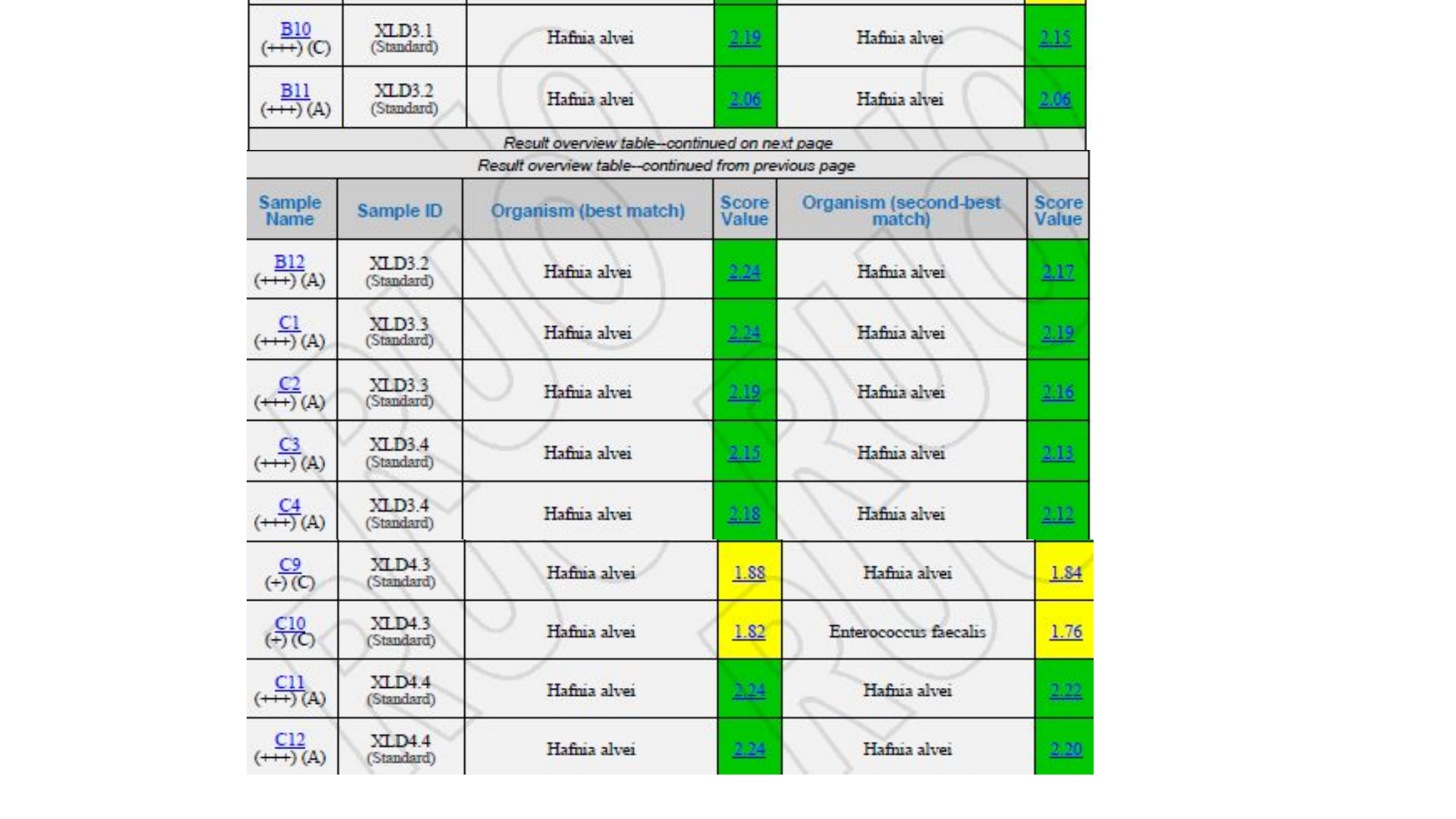

## Slide 2
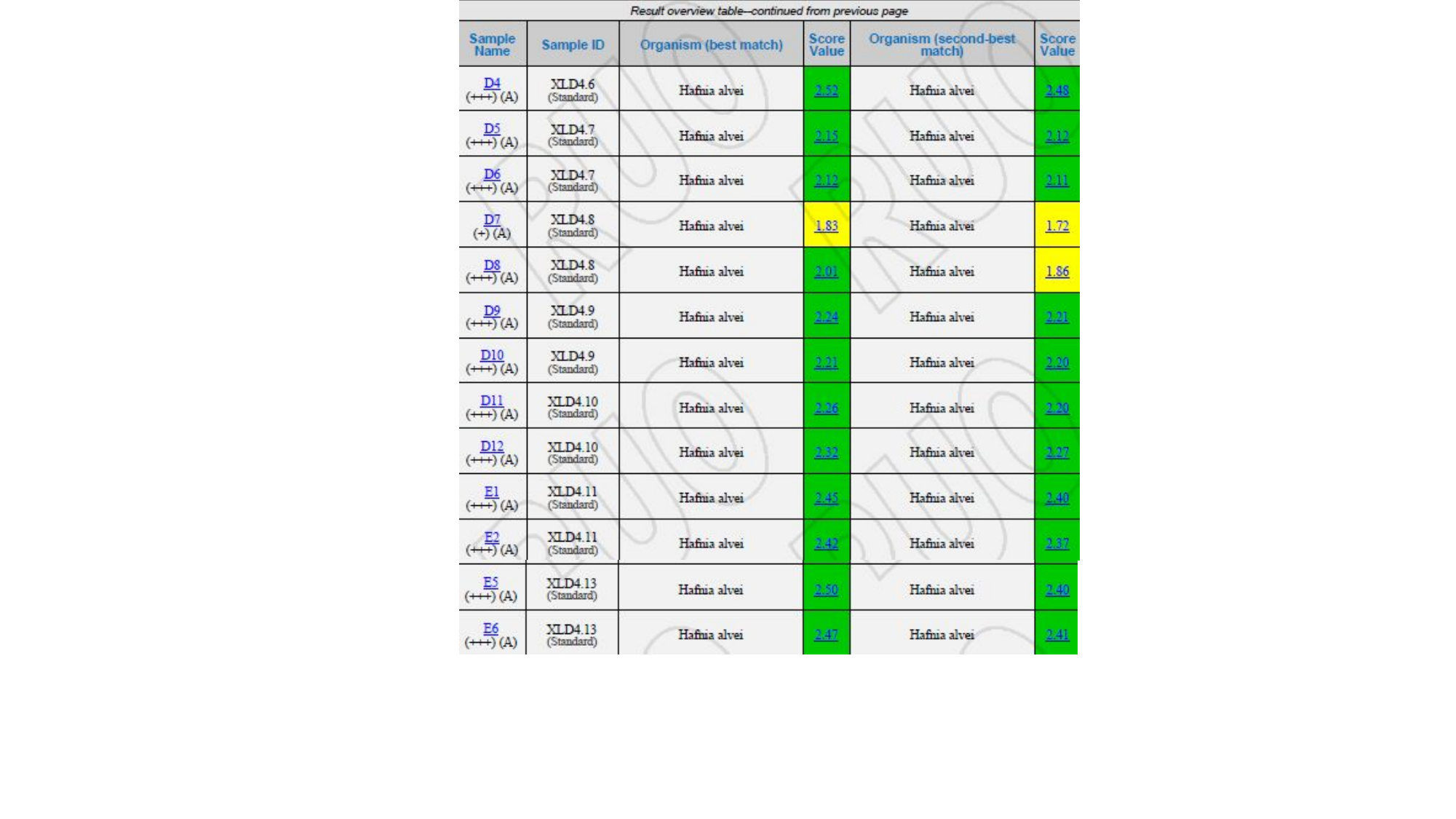

## Slide 3
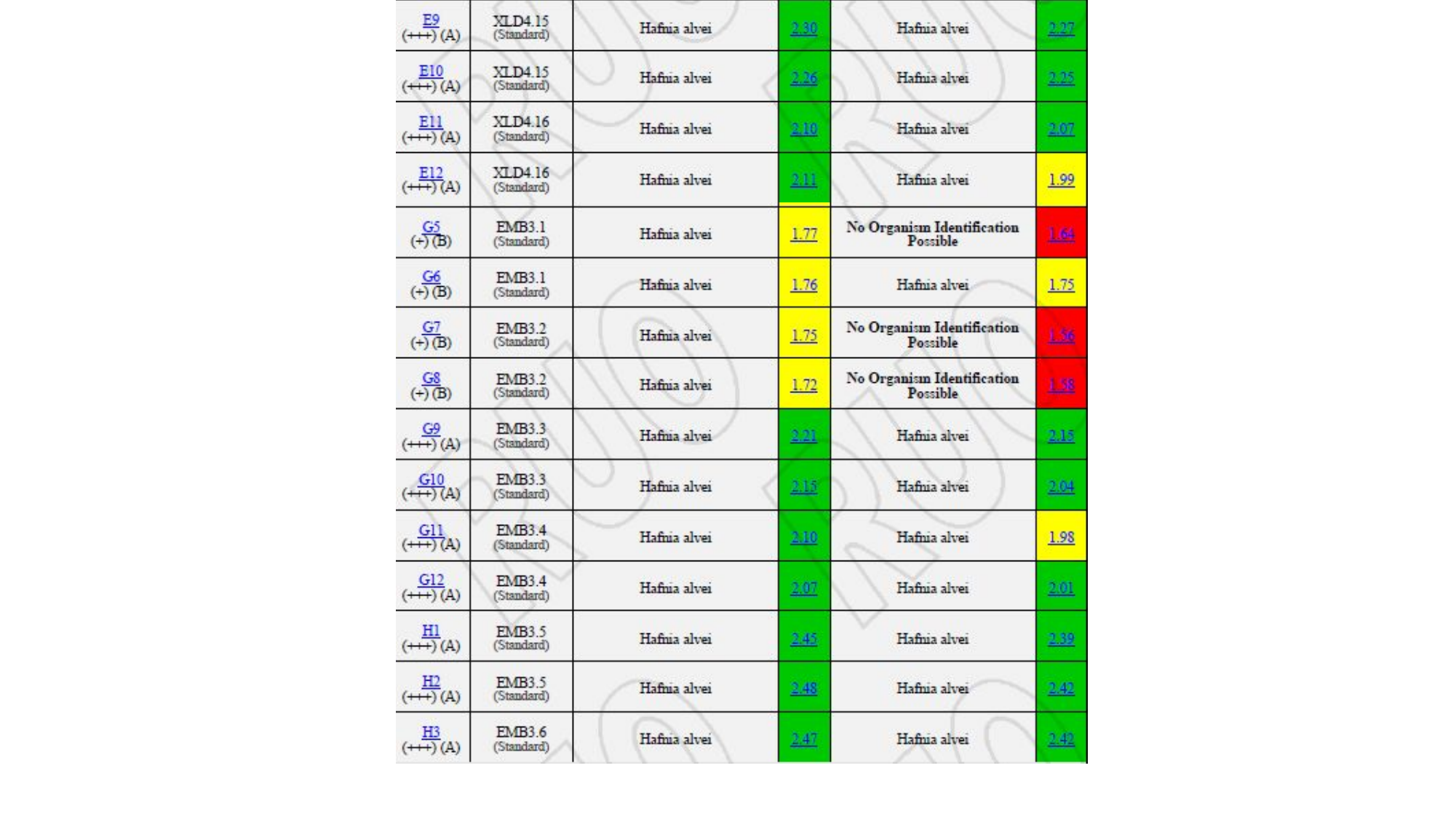

## Slide 4
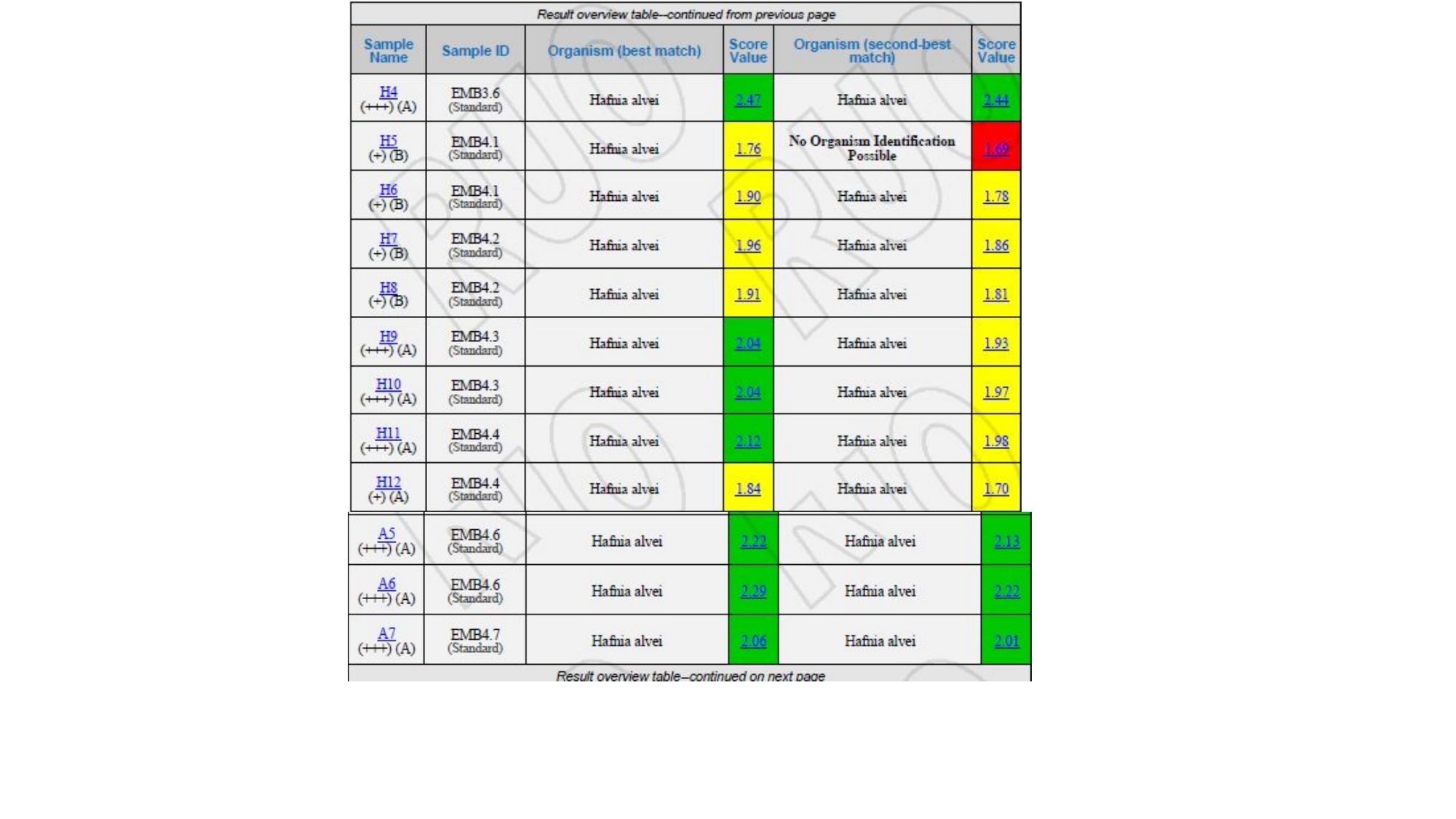

## Slide 5
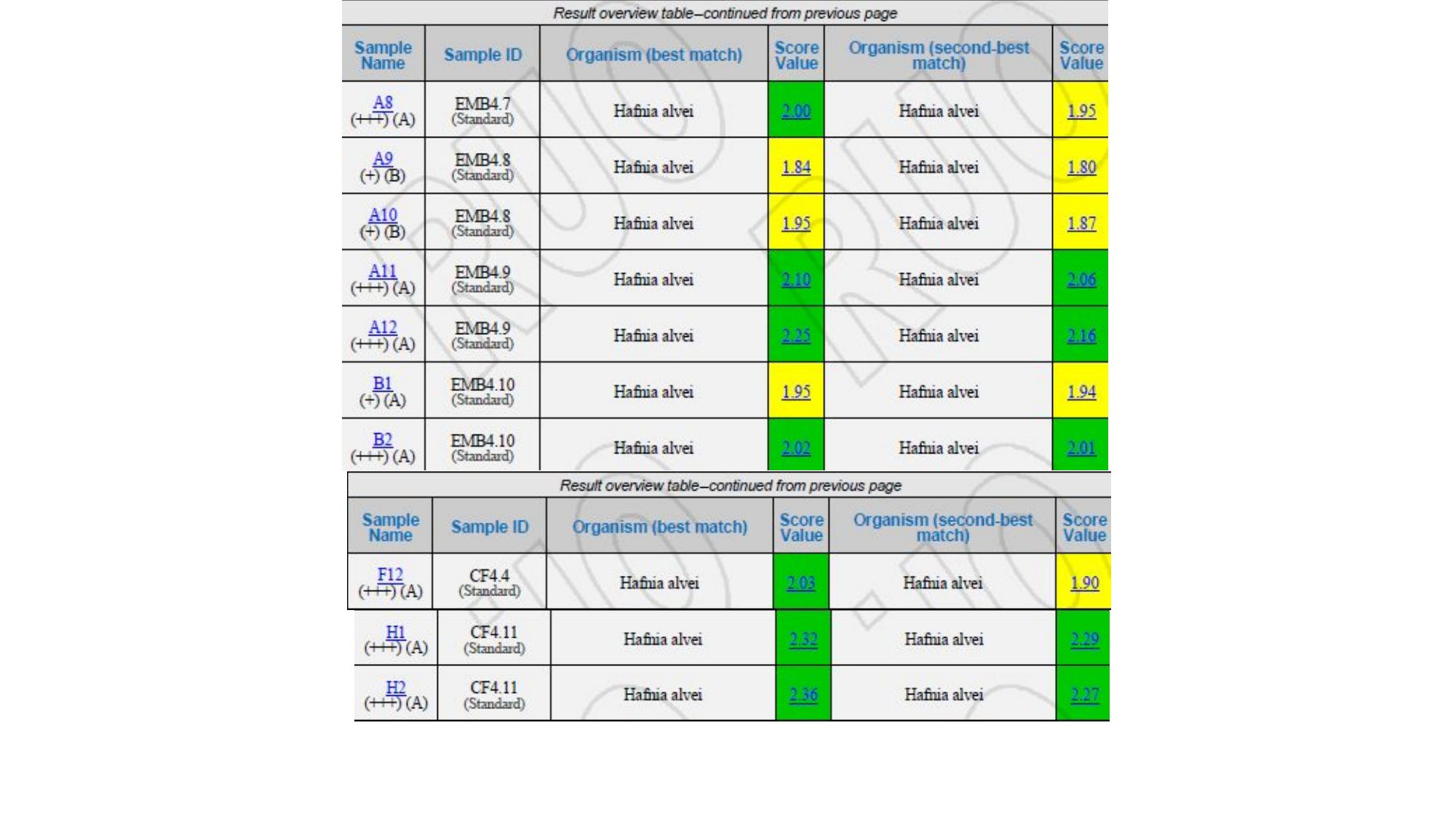

## Slide 6
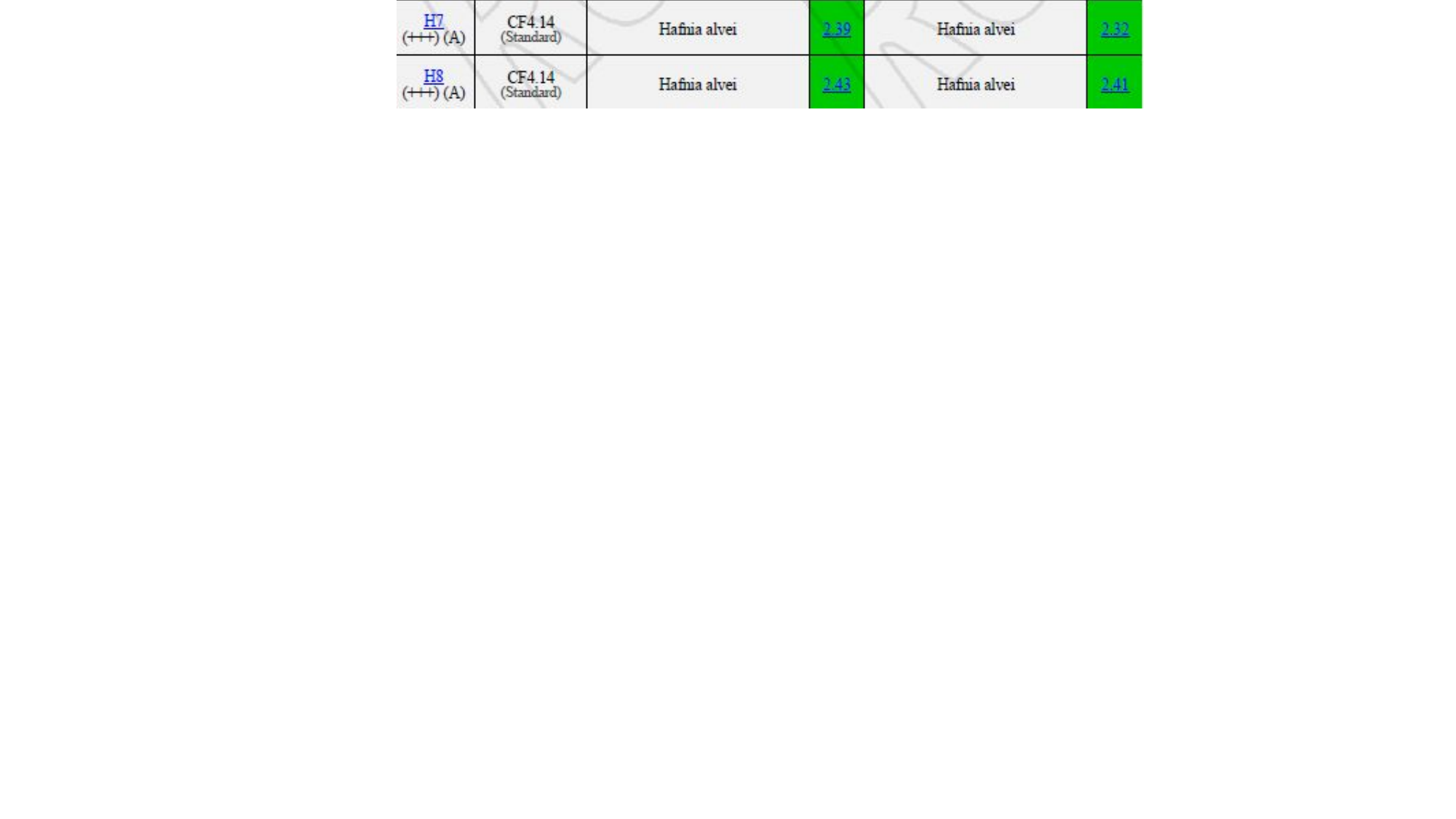

Supplement: Supplementary file 1 [file microorganisms-14-00741-s001.zip › Supplementary files for Hafnia.pptx]
